# Association of Mode of Obstetric Delivery With Child and Adolescent Body Composition

**DOI:** 10.1001/jamanetworkopen.2021.25161

**Published:** 2021-10-08

**Authors:** Lidia Mínguez-Alarcón, Sheryl L. Rifas-Shiman, Joanne E. Sordillo, Izzuddin M. Aris, Allison J. Wu, Marie-France Hivert, Emily Oken, Jorge E. Chavarro

**Affiliations:** 1 Department of Medicine, Harvard Medical School and Brigham and Women’s Hospital, Boston, Massachusetts; 2 Department of Environmental Health, Harvard School of Public Health, Boston, Massachusetts; 3 Department of Nutrition, Harvard School of Public Health, Boston, Massachusetts; 4 Department of Population Medicine, Harvard Medical School and Harvard Pilgrim Health Care Institute, Boston, Massachusetts; 5 Department of Obstetrics and Gynecology and Division of Chronic Disease Research Across the Lifecourse, Department of Population Medicine, Harvard Medical School and Harvard Pilgrim Health Care Institute, Boston, Massachusetts; 6 Division of Gastroenterology, Hepatology and Nutrition, Boston Children’s Hospital, Boston, Massachusetts; 7 Diabetes Unit, Massachusetts General Hospital, Boston; 8 Department of Epidemiology, Harvard School of Public Health, Boston, Massachusetts

## Abstract

**Importance:**

Although the literature on the association between birth by cesarean delivery and children’s anthropometry has continued to increase, only a few studies have examined the association of cesarean delivery with measures of body composition assessed using dual-energy x-ray absorptiometry (DXA), which allows the differentiation of fat and lean mass overall and in specific regions of the body.

**Objective:**

To investigate whether differences exist in DXA-measured body composition between children and adolescents born by cesarean delivery and those born by vaginal delivery.

**Design, Setting, and Participants:**

This prospective cohort study included singleton children of mothers enrolled between April 1999 and July 2002 in Project Viva, a longitudinal prebirth cohort of mother-child pairs in Massachusetts. The children had at least 1 DXA scan at a follow-up visit during middle childhood (2007-2010) and/or early adolescence (2013-2016). Data analysis was performed from October 16, 2020, to May 9, 2021.

**Exposures:**

Mode of delivery (cesarean vs vaginal).

**Main Outcomes and Measures:**

Total lean mass index, total and truncal fat mass indexes, visceral adipose tissue (VAT), subcutaneous abdominal adipose tissue, and total abdominal adipose tissue (TAAT) were estimated using DXA. Multivariable linear regression models were used to estimate the association between mode of delivery and DXA-derived outcomes with adjustment for confounders. Stabilized inverse probability weights were used to control for potential selection bias owing to loss to follow-up.

**Results:**

A total of 975 mother-child pairs were included in the study. The mean (SD) maternal age at study entry was 32.0 (5.5) years, and the mean (SD) self-reported prepregnancy body mass index (BMI; calculated as weight in kilograms divided by height in meters squared) was 25.0 (5.4). Of the children included in the study, 491 (50%) were female; 212 (22%) were born by cesarean delivery and 763 (78%) by vaginal delivery. Body composition in middle childhood as measured by DXA did not differ by mode of delivery. In early adolescence, participants born by cesarean delivery had a significantly greater total lean mass index (β, 0.4; 95% CI, 0.0-0.7), total fat mass index (β, 0.6; 95% CI, 0.1-1.1), truncal fat mass index (β, 0.3; 95% CI, 0.0-0.5), VAT area (β, 4.7; 95% CI, 0.9-8.6), and TAAT area (β, 23.8; 95% CI, 0.8-46.8) in a model adjusted for child sex and age at the time of DXA measurements; maternal age, educational level, race and ethnicity, total gestational weight gain, and smoking status during pregnancy; birth-weight-per-gestational-age *z* score; and paternal BMI. Associations between mode of delivery and measures of adiposity were found for cesarean deliveries performed in the absence of labor (total fat mass index: β, 1.3; 95% CI, 0.3-2.3; truncal fat mass index: β, 0.6; 95% CI, 0.1-1.0; VAT area: β, 10.7; 95% CI, 3.1-18.3; TAAT area: β, 47.3; 95% CI, 2.3-92.2). There were no associations after adjustment for maternal self-reported prepregnancy BMI (total lean mass index: β, 0.2; 95% CI, –0.1 to 0.6; total fat mass index: β, 0.4; 95% CI, –0.1 to 0.9; truncal fat mass index: β, 0.2; 95% CI, –0.1 to 0.4; VAT area: β, 3.0; 95% CI, –0.6 to 6.7; TAAT area: β, 13.6; 95% CI, –8.2 to 35.3).

**Conclusions and Relevance:**

In this cohort study, adolescents born by cesarean delivery had significantly higher measures of lean mass, fat mass, and central adiposity compared with those born by vaginal delivery, but associations did not remain after adjustment for the mothers’ self-reported prepregnancy BMI. The findings suggest that the association between birth by cesarean delivery and adolescent adiposity may partly be explained by maternal self-reported prepregnancy BMI.

## Key Points

**Question:**

Are there differences in body composition measured by dual-energy x-ray absorptiometry during middle childhood and early adolescence among children born by cesarean delivery vs vaginal delivery?

**Findings:**

In this cohort study of 975 mother-child pairs, adolescents born by cesarean delivery had significantly higher measures of lean mass, fat mass, and central adiposity compared with those born by vaginal delivery, but associations did not remain after adjustment for the mothers’ self-reported prepregnancy body mass index.

**Meaning:**

The findings suggest that prepregnancy body mass index may partly explain the association between birth by cesarean delivery and adiposity in adolescence.

## Teaser

This cohort study investigates associations between the mode of obstetric delivery and body composition measured by dual-energy x-ray absorptiometry among offspring during middle childhood and early adolescence.

## Introduction

Cesarean deliveries are the most common inpatient surgical procedure in the US,^1^ accounting for approximately one-third of deliveries nationwide.^2,3^ Cesarean delivery is associated with reduced mortality and serious morbidity to the mother and fetus when medically indicated.^4^ Nonetheless, it has been shown that women who undergo a low-risk planned cesarean delivery have an increased risk of major maternal morbidity, including cardiac arrest, emergency hysterectomy, and thromboembolism, compared with women who have a low-risk planned vaginal delivery.^5^ Furthermore, a higher frequency of respiratory complications and, in conjunction, higher rates of neonatal intensive care unit admissions have been observed among neonates born by cesarean delivery compared with those born by vaginal delivery.^6,7,8^

In addition to the short-term risks to mother and offspring, increasing evidence has shown that children born by cesarean delivery may also experience higher rates of adverse health outcomes later in life,^9^ including type 1 diabetes,^10^ allergies and asthma,^11,12,13^ multiple sclerosis,^14^ and chronic immune disorders.^15,16^ Although not observed in all studies,^17,18,19^ some studies,^20,21,22,23^ including 3 meta-analyses,^24,25,26^ have found a higher risk of obesity among children born by cesarean delivery. However, not all studies considered the common factors associated with mode of delivery and offspring body mass index (BMI), such as maternal prepregnancy BMI. A previous study^27^ using the Project Viva cohort, a longitudinal prebirth cohort of mother-offspring pairs in eastern Massachusetts, reported that compared with children born by vaginal delivery, children born by cesarean delivery had a higher BMI *z* score during childhood and early adolescence. Among participants of the Growing Up Today Study, there was a 15% (95% CI, 6%-26%) higher risk of obesity from late childhood through early adulthood among children born by cesarean delivery^20^ even after adjusting for maternal prepregnancy BMI.

In addition, although the literature on the association between birth by cesarean delivery and children’s anthropometric measures has continued to increase, only a few studies^28,29,30^ have examined the association of cesarean delivery with measures of body composition using dual-energy x-ray absorptiometry (DXA), which allows for the differentiation of fat and lean mass overall and in specific regions of the body. In children, anthropometric measures are difficult to interpret because they reflect not only differences in body composition but also differences in height, pubertal status, and rate of growth. In adults, anthropometric measures reflect adiposity more directly because height does not materially change. Therefore, we aimed to examine differences in DXA-measured body composition during middle childhood and early adolescence by the mode of delivery at birth among Project Viva participants. We hypothesized that children born by cesarean delivery would have a greater proportion of total and truncal fat than would those born by vaginal delivery.

## Methods

### Study Participants

In this cohort study, we enrolled participants into Project Viva between April 1999 and July 2002. The institutional review board of Harvard Pilgrim Health Care approved the study protocol.^31^ All mothers provided written informed consent, and children provided verbal assent at follow-up visits. The study population, enrollment, and follow-up procedures are described elsewhere.^31^ In brief, we recruited women attending their initial prenatal visit (at a median of 9.9 weeks of gestation) at Atrius Harvard Vanguard Medical Associates, a multispecialty group practice. Eligibility criteria included fluency in English, a singleton pregnancy, a gestational age of 22 weeks or less at recruitment, and no plans to move away from the study area before delivery. A trained research assistant conducted in-person study visits at the end of the first and second trimesters of pregnancy and with both mother and child after delivery and during middle childhood (2007-2010) and early adolescence (2013-2016). We performed the data analysis from October 16, 2020, to May 9, 2021. We collected data from multiple sources, including interviews and surveys, medical records, examinations, and biospecimens. This study followed the Strengthening the Reporting of Observational Studies in Epidemiology (STROBE) reporting guideline.

### Mode of Delivery

We obtained information about mode of delivery from electronic hospital records. For each participant who had a cesarean delivery recorded on electronic birth logs, we reviewed the operative report to confirm cesarean delivery and to abstract the primary indication for operative delivery and the timing of cesarean delivery in relation to the onset of labor. Using this information, we divided cesarean deliveries into unplanned and planned cesarean deliveries. We defined an unplanned cesarean delivery as a delivery in which the operative report described a failed induction of labor, prolonged latent phase, prolonged active phase, arrest of dilation, failure to progress, arrest of descent in the second stage of labor, failed operative vaginal delivery, nonreassuring fetal heart rate tracing, nonreassuring testing prompting immediate cesarean delivery, umbilical cord prolapse, or placental abruption.^23^ All unplanned cesarean deliveries were preceded by labor. We defined planned cesarean deliveries as those for which participants did not undergo a trial of labor (including an elective repeated cesarean delivery without a trial of labor and pregnancies in which malpresentation, placenta previa, suspected macrosomia, maternal request, or another indication precluded trial of labor).

### Body Composition Measurements

At the middle childhood and early adolescence in-person visits, total lean mass and total and truncal fat mass as well as visceral adipose tissue (VAT), subcutaneous abdominal adipose tissue (SAAT), and total abdominal adipose tissue (TAAT) were estimated using DXA. Research assistants administered whole-body DXA scans with the Discovery DXA System (Hologic Inc). A trained research assistant checked all scans for positioning, movement, and artifacts. We used the same DXA machine for all participants at both visits. We used DXA Hologic APEX software, version 4.0, to obtain the total lean mass index (calculated as lean mass divided by height in meters squared), total and truncal fat mass index (calculated as fat mass divided by height in meters squared), SAAT area, and TAAT area. The research assistant reviewed all analyzed scans to confirm or correct accurate placement of the software’s automatically generated reference lines (intrarater reliability between the software and the research assistant: *r*, 0.99). As previously described,^32^ the Hologic software estimates the VAT area by subtracting the SAAT area from the TAAT area in the abdominal region at approximately the fourth or fifth lumbar vertebrae, with the bottom edge situated at 1 cm above the iliac crest.

### Covariates

Mothers reported their age, race and ethnicity, educational level, parity, and prepregnancy weight and height and father’s weight and height, and we calculated the BMI (calculated as weight in kilograms divided by height in meters squared) for both the mothers and the fathers. We calculated the mother’s gestational weight gain by subtracting the self-reported prepregnancy weight from the last clinically recorded prenatal weight. We obtained the child’s birth weight and delivery date from hospital medical records. We calculated gestational age at birth using the date of the last menstrual period. If the estimate of gestational age by the second-trimester ultrasonography differed from the calculated gestational age by more than 10 days, we used the date of the ultrasonography. We calculated the pubertal development score using a 5-item written pubertal development scale^33^ completed by the parent at the early adolescent visit. Items included on the boys’ pubertal development scale were voice deepening, body hair growth, facial hair growth, acne, and growth spurt. Items included on the girls’ pubertal development scale were breast development, body hair growth, acne, growth spurt, and menarche. Response options and scores for each pubertal development scale item except menarche were the following: 1, “not yet started”; 2, “barely started”; 3, “definitely started”; and 4, “seems complete.” We coded menarche as 4 points if menarche was present and 1 point if not present. To assign a summary pubertal development score to each participant, we calculated the mean score across all items.^34^

### Statistical Analysis

Participants’ characteristics and DXA-derived body composition and adiposity outcomes for the overall study population and by mode of obstetric delivery are presented as either means and SDs or numbers and percentages. Main analyses were performed using the mode of delivery as a 2-category variable: cesarean or vaginal delivery. In secondary analyses, we used data on the timing and type of labor in relation to the mode of delivery and on planned cesarean deliveries and conducted separate analyses defining mode of delivery as a 3-category variable: planned cesarean delivery (without trial of labor), unplanned cesarean delivery (after spontaneous or induced labor), or vaginal delivery. We used multivariable linear regression models to estimate the association between mode of delivery and DXA-derived body composition outcomes during middle childhood and early adolescence with adjustment for confounders associated with both mode of delivery and body composition outcomes. We presented 4 models: model 0 was unadjusted; model 1 was adjusted for the child’s sex and age at the time of the DXA measurements and for the mother’s age, educational level, and race and ethnicity; model 2 was adjusted for all model 1 variables plus the mother’s total gestational weight gain, her smoking status during pregnancy, the child’s *z* score for birth weight per gestational age, and the paternal BMI; and model 3 was adjusted for all model 2 variables plus the maternal prepregnancy BMI. We used stabilized inverse probability weights to control for potential selection bias owing to loss to follow-up at either the middle childhood or early adolescence visits.^35^ The denominator of the inverse probability weights was the probability of remaining uncensored (ie, not lost to follow-up), which was conditional on the exposure and the following covariates: the mother’s age, educational level, race and ethnicity, self-reported prepregnancy BMI, total gestational weight gain, and smoking status; the mode of delivery; the child’s *z* score for birth weight per gestational age; and the paternal BMI. The numerator was the same as its denominator counterpart but without conditioning on covariates. Results are reported as β (95% CI). All statistical analyses were performed using SAS, version 9.4 (SAS Institute Inc).

## Results

Of 2128 live singleton infants, data on mode of delivery were available for 2098. This study included 975 children who had at least 1 DXA scan performed during the middle childhood (876 participants; median age, 7.7 years [IQR, 7.3-8.2 years]) or early adolescence (740 participants; median age, 12.8 years [IQR, 12.5-13.2 years]) study visits. Compared with the excluded participants, those included had similar rates of cesarean delivery (excluded: 22%; included: 25%) and participant characteristics, including maternal age, prepregnancy BMI, race and ethnicity, educational level, and smoking status; paternal BMI; and sex of the child (eTable in the Supplement).

At study entry, the mean (SD) maternal age was 32.0 (5.5) years, and the mean (SD) prepregnancy BMI was 25.0 (5.4) (Table 1). Of the children included in the study, 491 (50%) were female; 212 (22%) were born by cesarean delivery and 763 (78%) by vaginal delivery. Compared with women who delivered vaginally, women who delivered by cesarean were older (mean [SD] age, 32.6 [5.0] years vs 31.8 [5.6] years), had a greater mean (SD) prepregnancy BMI (26.3 [6.3] vs 24.7 [5.0]), and were less likely to be White individuals (126 [60%] vs 505 [67%]). Compared with children born vaginally, those born by cesarean delivery had a higher mean (SD) birth-weight-for-gestational-age *z* score (0.31 [1.09] units vs 0.16 [0.94] units). No other maternal, paternal, or offspring characteristics substantially differed by mode of delivery (Table 1).

**Table 1.** Characteristics of 975 Mother-Child Pairs With DXA Outcomes in Middle Childhood or Early Adolescence by Mode of Obstetric Delivery in Project Viva

Characteristic	Overall	Vaginal delivery	Cesarean delivery
Mother-child pairs, No. (%)	975 (100)	763 (78)	212 (22)
Mother			
Age, mean (SD), y	32.0 (5.5)	31.8 (5.6)	32.6 (5.0)
GA at enrollment, mean (SD), wk	.5 (2.6)	10.5 (2.6)	10.3 (2.6)
Prepregnancy BMI, mean (SD)	25.0 (5.4)	24.7 (5.0)	26.3 (6.3)
Total GWG, mean (SD), kg	15.4 (5.5)	15.5 (5.4)	15.4 (5.9)
Nulliparous, No. (%)			
No	521 (53)	420 (55)	101 (48)
Yes	454 (47)	343 (45)	111 (52)
Race and ethnicity, No. (%)			
Black	175 (18)	131 (17)	44 (21)
Hispanic	65 (7)	50 (7)	15 (7)
White	631 (65)	505 (67)	126 (60)
Other^a^	98 (10)	72 (9)	26 (12)
College graduate, No. (%)			
No	326 (34)	263 (35)	63 (30)
Yes	643 (66)	495 (65)	148 (70)
Pregnancy smoking status, No. (%)			
Never	694 (71)	541 (71)	153 (73)
Former	180 (19)	142 (19)	38 (18)
Current	97 (10)	77 (10)	20 (9)
Paternal BMI, mean (SD)	26.5 (4.0)	26.4 (3.9)	27.0 (4.2)
Child			
Sex, No. (%)			
Male	484 (50)	377 (49)	107 (50)
Female	491 (50)	386 (51)	105 (50)
GA at delivery, mean (SD), wk	39.6 (1.8)	39.6 (1.7)	39.4 (2.1)
Birth weight for GA *z* score, mean (SD)	0.19 (0.98)	0.16 (0.94)	0.31 (1.09)
Middle childhood visit			
Age, median (IQR), y	7.7 (7.3-8.2)	7.7 (7.3-8.2)	7.6 (7.3-8.1)
Height, mean (SD), cm	128.4 (7.5)	128.4 (7.6)	128.2 (7.5)
BMI, mean (SD)	17.3 (3.1)	17.2 (2.9)	17.7 (3.4)
CDC BMI *z* score, mean (SD)	0.42 (1.00)	0.37 (0.99)	0.58 (1.01)
Early adolescent visit			
Age, median (IQR), y	12.8 (12.5-13.2)	12.8 (12.5-13.2)	12.7 (12.4-13.2)
Pubertal development score, median (IQR)	2.6 (2.0-3.2)	2.6 (2.0-3.2)	2.6 (1.8-3.2)
Height, mean (SD), cm	159.1 (8.6)	159.2 (8.6)	158.8 (8.7)
BMI, mean (SD)	21.0 (4.7)	20.7 (4.5)	21.8 (5.2)
CDC BMI *z* score, mean (SD)	0.41 (1.07)	0.36 (1.06)	0.61 (1.09)

Abbreviations: BMI, body mass index (calculated as weight in kilograms divided by height in meters squared); CDC, Centers for Disease Control and Prevention; DXA, dual-energy x-ray absorptiometry; GA, gestational age; GWG, gestational weight gain.

^a^ Other included American Indian, Alaska Native, or Pacific Islander.

The mean DXA-derived body composition outcomes during middle childhood were similar for children born by cesarean delivery and those born vaginally; however, compared with adolescents born by vaginal delivery, those born by cesarean delivery had greater total lean mass index (mean [SD], 15.3 [2.2] vs 14.8 [2.1]), total and truncal fat mass index (mean [SD], 6.8 (3.6) vs 6.2 [3.0]), VAT area (mean [SD], 45.1 [26.1] vs 39.4 [22.5]), SAAT area (mean [SD], 176.2 [135.7] vs 161.5 [117.9]), and TAAT area (mean [SD], 221.4 [156.6] vs 200.8 [133.5]) (Table 2). Consistent with the crude comparisons, we found no difference in DXA adiposity measures during middle childhood by mode of delivery (Table 3 and Table 4). However, adolescents born by cesarean delivery had a significantly greater total lean mass index (β, 0.4; 95% CI, 0.0-0.7), total fat mass index (β, 0.6; 95% CI, 0.1-1.1), truncal fat mass index (β, 0.3; 95% CI, 0.0-0.5), VAT area (β, 4.7; 95% CI, 0.9-8.6), and TAAT area (β, 23.8; 95% CI, 0.8-46.8) in a model adjusted for the child’s sex and age at the time of DXA measurements; for maternal age, educational level, race and ethnicity, total gestational weight gain, and smoking status during pregnancy; for birth-weight-for-gestational-age *z* score; and for paternal BMI (Table 3).

**Table 2.** Dual-Energy X-ray Absorptiometry–Derived Body Composition Outcomes During Middle Childhood and Early Adolescence by Mode of Obstetric Delivery in Project Viva

Body composition outcome	Mean (SD)		
	Overall	Vaginal delivery	Cesarean delivery
**Middle childhood**			
Participants, No. (%)	876 (100)	683 (78)	193 (22)
Total lean mass index^a^	13.0 (1.5)	13.0 (1.4)	13.2 (1.5)
Total fat mass index^b^	4.4 (2.0)	4.4 (1.9)	4.6 (2.1)
Truncal fat mass index^b^	1.5 (0.9)	1.5 (0.9)	1.5 (0.9)
VAT area, cm^2^	26.6 (15.9)	26.5 (15.6)	26.6 (16.9)
SAAT area, cm^2^	104.8 (73.2)	104.9 (72.6)	104.6 (75.3)
TAAT area, cm^2^	131.4 (80.5)	131.4 (79.6)	131.2 (83.8)
**Early adolescence**			
Participants, No. (%)	740 (100)	583 (79)	157 (21)
Total lean mass index^a^	14.9 (2.1)	14.8 (2.1)	15.3 (2.2)
Total fat mass index^b^	6.3 (3.1)	6.2 (3.0)	6.8 (3.6)
Truncal fat mass index^b^	2.4 (1.5)	2.4 (1.4)	2.6 (1.7)
VAT area, cm^2^	40.6 (23.4)	39.4 (22.5)	45.1 (26.1)
SAAT area, cm^2^	164.6 (121.9)	161.5 (117.9)	176.2 (135.7)
TAAT area, cm^2^	205.2 (138.8)	200.8 (133.5)	221.4 (156.6)

Abbreviations: SAAT, subcutaneous abdominal adipose tissue; TAAT, total abdominal adipose tissue; VAT, visceral adipose tissue.

^a^ Calculated as lean mass divided by height in meters squared.

^b^ Calculated as fat mass divided by height in meters squared.

**Table 3.** Differences in Dual-Energy X-ray Absorptiometry–Assessed Body Composition Metrics During Middle Childhood and Early Adolescence Between Children Born by Cesarean Delivery vs Vaginal Delivery

Metric	β (95% CI)^a^			
	Model 0^b^	Model 1^c^	Model 2^d^	Model 3^e^
**Middle childhood (n = 876)**				
Total lean mass index	0.1 (–0.1 to 0.4)	0.2 (0.0-0.4)	0.1 (–0.1 to 0.3)	0.1 (–0.1 to 0.3)
Total fat mass index	0.2 (–0.1 to 0.5)	0.2 (–0.1 to 0.5)	0.2 (–0.1 to 0.4)	0.0 (–0.2 to 0.3)
Truncal fat mass index	0.0 (–0.1 to 0.2)	0.1 (–0.1 to 0.2)	0.0 (–0.1 to 0.2)	0.0 (–0.1 to 0.1)
VAT area	–0.2 (–2.8 to 2.4)	0.6 (–1.6 to 2.7)	0.0 (–2.1 to 2.0)	–0.7 (–2.7 to 1.4)
SAAT area	0.1 (–11.7 to 11.9)	1.6 (–7.8 to 10.9)	–0.7 (–9.7 to 8.3)	–3.9 (–12.7 to 4.9)
TAAT area	–0.1 (–13.0 to 12.8)	2.1 (–9.1 to 13.3)	–0.7 (–11.5 to 10.1)	–4.6 (–15.2 to 5.9)
**Early adolescence (n = 740)**				
Total lean mass index	0.6 (0.2-0.9)	0.5 (0.1-0.8)	0.4 (0.0-0.7)	0.2 (–0.1 to 0.6)
Total fat mass index	0.7 (0.2-1.3)	0.8 (0.2-1.3)	0.6 (0.1-1.1)	0.4 (–0.1 to 0.9)
Truncal fat mass index	0.3 (0.0-0.6)	0.3 (0.1-0.6)	0.3 (0.0-0.5)	0.2 (–0.1 to 0.4)
VAT area	6.5 (2.2-10.7)	5.9 (1.8-9.9)	4.7 (0.9-8.6)	3.0 (–0.6 to 6.7)
SAAT area	19.2 (–3.0 to 41.4)	25.0 (4.4-45.5)	19.1 (–0.5 to 38.8)	10.6 (–8.1 to 29.2)
TAAT area	25.7 (0.4-50.9)	30.9 (6.8-55.0)	23.8 (0.8-46.8)	13.6 (–8.2 to 35.3)

Abbreviations: SAAT, subcutaneous abdominal adipose tissue; TAAT, total abdominal adipose tissue; VAT, visceral adipose tissue.

^a^ The reference group was children born by vaginal delivery. Stabilized inverse probability weights were used to control for potential selection bias owing to loss to follow-up at either the middle childhood or early adolescence visits.

^b^ Unadjusted.

^c^ Adjusted for child age and sex at the time of dual-energy x-ray absorptiometry measurements and maternal age, educational level, and race and ethnicity.

^d^ Adjusted for all model 1 criteria plus total gestational weight gain, maternal smoking status during pregnancy, birth-weight-for-gestational-age *z* score, and paternal body mass index, calculated as weight in kilograms divided by height in meters squared.

^e^ Adjusted for all model 2 criteria plus prepregnancy maternal body mass index.

**Table 4.** Differences in Dual-Energy X-ray Absorptiometry–Assessed Body Composition Metrics During Middle Childhood and Early Adolescence Between Children Born by CD (Unplanned or Planned) vs Vaginal Delivery^a^

Metric	β (95% CI)^b^			
	Model 0^c^	Model 1^d^	Model 2^e^	Model 3^f^
**Middle childhood (n = 876)^g^**				
Total lean mass index				
Vaginal delivery	1 [Reference]	1 [Reference]	1 [Reference]	1 [Reference]
Unplanned CD	0.1 (–0.1 to 0.4)	0.1 (–0.1 to 0.4)	0.1 (–0.1 to 0.3)	0.0 (–0.2 to 0.3)
Planned CD	0.2 (–0.2 to 0.6)	0.3 (0.0 to 0.7)	0.2 (–0.1 to 0.6)	0.1 (–0.2 to 0.5)
Total fat mass index				
Vaginal delivery	1 [Reference]	1 [Reference]	1 [Reference]	1 [Reference]
Unplanned CD	0.2 (–0.2 to 0.5)	0.2 (–0.2 to 0.5)	0.1 (–0.2 to 0.4)	0.0 (–0.3 to 0.3)
Planned CD	0.2 (–0.3 to 0.8)	0.4 (–0.1 to 0.9)	0.3 (–0.2 to 0.8)	0.1 (–0.4 to 0.6)
Truncal fat mass index				
Vaginal delivery	1 [Reference]	1 [Reference]	1 [Reference]	1 [Reference]
Unplanned CD	0.0 (–0.1 to 0.2)	0.0 (–0.1 to 0.2)	0.0 (–0.1 to 0.2)	0.0 (–0.2 to 0.1)
Planned CD	0.1 (–0.2 to 0.3)	0.1 (–0.1 to 0.4)	0.1 (–0.1 to 0.3)	0.0 (–0.2 to 0.2)
VAT area				
Vaginal delivery	1 [Reference]	1 [Reference]	1 [Reference]	1 [Reference]
Unplanned CD	–0.7 (–3.7 to 2.3)	–0.2 (–2.7 to 2.3)	–0.7 (–3.1 to 1.7)	–1.2 (–3.6 to 1.1)
Planned CD	0.9 (–3.6 to 5.3)	2.2 (–1.5 to 5.9)	1.5 (–2.1 to 5.1)	0.5 (–3.1 to 4.0)
SAAT area				
Vaginal delivery	1 [Reference]	1 [Reference]	1 [Reference]	1 [Reference]
Unplanned CD	0.4 (–13.2 to 14.0)	–0.4 (–11.1 to 10.3)	–2.5 (–12.9 to 7.9)	–5.1 (–15.2 to 5.0)
Planned CD	–0.9 (–21.0 to 19.2)	6.1 (–9.9 to 22.2)	3.5 (–12.1 to 19.1)	–1.6 (–16.7 to 13.6)
TAAT area				
Vaginal delivery	1 [Reference]	1 [Reference]	1 [Reference]	1 [Reference]
Unplanned CD	–0.3 (–15.3 to 14.6)	–0.6 (–13.5 to 12.3)	–3.2 (–15.6 to 9.3)	–6.4 (–18.5 to 5.7)
Planned CD	0.0 (–22.0 to 22.0)	8.3 (–11.0 to 27.6)	5.0 (–13.7 to 23.6)	–1.1 (–19.3 to 17.1)
**Early adolescence (n = 740)** ^h^				
Total lean mass index				
Vaginal delivery	1 [Reference]	1 [Reference]	1 [Reference]	1 [Reference]
Unplanned CD	0.6 (0.1 to 1.0)	0.5 (0.1 to 0.9)	0.4 (0.0 to 0.8)	0.3 (–0.1 to 0.6)
Planned CD	0.5 (–0.2 to 1.2)	0.5 (–0.1 to 1.1)	0.4 (–0.3 to 1.0)	0.1 (–0.4 to 0.7)
Total fat mass index				
Vaginal delivery	1 [Reference]	1 [Reference]	1 [Reference]	1 [Reference]
Unplanned CD	0.6 (–0.1 to 1.2)	0.6 (0.0 to 1.3)	0.5 (–0.1 to 1.1)	0.3 (–0.3 to 0.8)
Planned CD	1.3 (0.3 to 2.3)	1.3 (0.3 to 2.3)	1.1 (0.2 to 2.1)	0.8 (–0.1 to 1.7)
Truncal fat mass index				
Vaginal delivery	1 [Reference]	1 [Reference]	1 [Reference]	1 [Reference]
Unplanned CD	0.2 (–0.1 to 0.5)	0.3 (0.0 to 0.6)	0.2 (–0.1 to 0.5)	0.1 (–0.2 to 0.4)
Planned CD	0.6 (0.1 to 1.0)	0.6 (0.1 to 1.0)	0.5 (0.1 to 1.0)	0.3 (–0.1 to 0.8)
VAT area				
Vaginal delivery	1 [Reference]	1 [Reference]	1 [Reference]	1 [Reference]
Unplanned CD	4.9 (0.0 to 9.8)	4.6 (–0.1 to 9.2)	3.4 (–1.0 to 7.8)	1.9 (–2.3 to 6.1)
Planned CD	10.7 (3.1 to 18.3)	9.5 (2.3 to 16.7)	8.5 (1.6 to 15.3)	6.1 (–0.4 to 12.6)
SAAT area				
Vaginal delivery	1 [Reference]	1 [Reference]	1 [Reference]	1 [Reference]
Unplanned CD	13.3 (–12.3 to 38.8)	20.0 (–3.6 to 43.5)	14.3 (–8.2 to 36.9)	6.6 (–14.7 to 27.9)
Planned CD	36.5 (–2.9 to 76.0)	42.2 (5.8 to 78.7)	36.9 (1.9 to 71.9)	25.0 (–8.1 to 58.1)
TAAT area				
Vaginal delivery	1 [Reference]	1 [Reference]	1 [Reference]	1 [Reference]
Unplanned CD	18.1 (–10.9 to 47.2)	24.5 (–3.1 to 52.2)	17.7 (–8.6 to 44.1)	8.5 (–16.4 to 33.4)
Planned CD	47.3 (2.3 to 92.2)	51.7 (8.9 to 94.6)	45.4 (4.4 to 86.4)	31.1 (–7.5 to 69.8)

Abbreviations: CD, cesarean delivery; SAAT, subcutaneous abdominal adipose tissue; TAAT, total abdominal adipose tissue; VAT, visceral adipose tissue.

^a^ Unplanned CDs were preceded by labor, and planned CDs were not preceded by labor.

^b^ The reference group was children born by vaginal delivery. Stabilized inverse probability weights were used to control for potential selection bias owing to loss to follow-up at either the middle childhood or early adolescence visits.

^c^ Unadjusted.

^d^ Adjusted for child age and sex at the time of dual-energy x-ray absorptiometry measurements and maternal age, educational level, and race and ethnicity.

^e^ Adjusted for all model 1 criteria plus total gestational weight gain, maternal smoking status during pregnancy, birth-weight-for-gestational-age *z* score, and paternal body mass index, calculated as weight in kilograms divided by height in meters squared.

^f^ Adjusted for all model 2 criteria plus prepregnancy maternal body mass index.

^g^ The middle childhood category included 683 vaginal deliveries, 131 unplanned CDs, 60 planned CDs, and 2 cases with missing values.

^h^ The early adolescence category included 583 vaginal deliveries, 108 unplanned CDs, 46 planned CDs, and 3 cases with missing values.

When we divided cesarean deliveries into planned and unplanned categories, associations between cesarean delivery and DXA-measured adiposity were found among adolescents born by planned cesarean delivery compared with adolescents born vaginally for total fat mass index (β, 1.3; 95% CI, 0.3-2.3), truncal fat mass index (β, 0.6; 95% CI, 0.1-1.0), VAT area (β, 10.7; 95% CI, 3.1-18.3), and TAAT area (β, 47.3; 95% CI, 2.3-92.2) in the unadjusted model (Table 4). For unplanned cesarean delivery compared with vaginal delivery, there were associations with total lean mass index (β, 0.6; 95% CI, 0.1-1.0) and VAT area (β, 4.9; 95% CI, 0.0-9.8) in the unadjusted model (Table 4). In the model including adjustment for maternal prepregnancy BMI, there was no association between cesarean delivery and total lean mass index (β, 0.2; 95% CI, –0.1 to 0.6), total fat mass index (β, 0.4; 95% CI, –0.1 to 0.9), truncal fat mass index (β, 0.2; 95% CI, –0.1 to 0.4), VAT area (β, 3.0; 95% CI, –0.6 to 6.7), and TAAT area (β, 13.6; 95% CI, –8.2 to 35.3) (Table 3 and Table 4).

## Discussion

In this prospective cohort study, we evaluated the associations between the mode of obstetric delivery and DXA-derived adiposity during middle childhood and early adolescence. Although we did not observe any associations during middle childhood, we found that adolescents born by cesarean delivery had higher lean mass and total and truncal fat mass indexes and greater VAT and TAAT areas than did those born by vaginal delivery. Differences in adiposity but not in lean mass were significantly greater for adolescents born by cesarean delivery without labor compared with those born by vaginal delivery in all models except the one adjusted for prepregnancy BMI. Thus, differences in prepregnancy maternal BMI appeared to at least partly account for these differences. The study’s results suggest the need for further research of body composition in children using direct assessments that can differentiate lean from fat mass and allow estimation of regional adiposity. The study’s results also suggest a need to account for important maternal confounders when investigating associations between the mode of obstetric delivery and health outcomes among offspring.

The results are consistent with those of 3 previous studies^28,29,30^ investigating cesarean delivery and DXA-derived body composition outcomes in children and adolescents while accounting for maternal confounders such as prepregnancy BMI. However, none of those studies explored subcategories of cesarean delivery (type of labor) and their association with DXA outcomes. In a birth cohort study from Brazil,^28^ the authors found that participants who were born by cesarean delivery had a higher fat mass index 6, 18, and 30 years later in life compared with those born by vaginal delivery; however, these associations were found only in crude analyses and not after controlling for confounders including prepregnancy BMI. Similarly, the other 2 studies,^29,30^ which also adjusted for maternal BMI, found no associations between mode of delivery and adiposity using DXA among neonates, children, and teenagers (aged 6 months to 19 years). Of interest, in a recent study^19^ including 15 069 children in the Promotion of Breastfeeding Intervention Trial (PROBIT) cohort in Belarus, the authors found that cesarean delivery was associated with greater child BMI, sum of skinfolds, percentage of body fat, and fat mass index at 6.5 and 11.5 years; however, further adjustment for maternal BMI resulted in no associations. The current study’s findings are not consistent with those of the preponderance of the current literature^20,21,22,23,24,25,26^ examining the association between birth by cesarean delivery and childhood obesity. Of note, a previous study by Mueller et al^27^ reported that infants born by cesarean delivery had higher BMI *z* scores during childhood and early adolescence compared with infants born by vaginal delivery, even after adjusting for maternal prepregnancy BMI. It is important to note the differences between that study and the current one. First, in the study by Mueller et al,^27^ the association of cesarean delivery with BMI *z* score persisted regardless of the type of labor and was noted slightly more among children than adolescents, whereas in the current study, more associations between cesarean delivery and measures of adiposity were noted in early adolescence and for cesarean deliveries performed without labor. Second, the current study included a smaller sample size than did the study by Mueller et al,^27^ which resulted in similar but more modest effect estimates for results for early adolescence compared with the findings of that study. The differences by childhood period between the current study and the study by Mueller et al^27^ were based on anthropometric measurements and suggest some limitations of using anthropometric measurements to assess body composition in children. Whereas BMI (and BMI *z* score in children) is widely used in clinical and research settings and is associated with measures of adiposity in adults and children, more associations have been found between BMI and DXA measures of adiposity in adults than in children.^36,37^ Dual-energy x-ray absorptiometry has advantages over traditional anthropometric measures, such as the assessment of regional adiposity, and has more subtle advantages among children. Unlike in adults, for whom height is fixed and BMI is unrelated to height (thus allowing anthropometric measurements to reflect adiposity more directly), anthropometric indexes for children reflect not only differences in body composition but also differences in height, pubertal status, and rate of growth, making it more difficult to interpret anthropometric measures for children. This study’s findings suggest that for research questions in which differences between groups may be modest, the use of DXA may be preferable to the use of anthropometric measures.

This study’s results also suggest the importance of accounting for maternal prepregnancy and pregnancy characteristics in studies investigating the association of events during pregnancy with children’s health in general. Although multiple previous studies,^20,21,22,23^ including systematic reviews and meta-analyses,^24,25,26^ have suggested an association between birth by cesarean delivery and childhood obesity, a common limitation in the existing literature is the lack of adjustment for maternal prepregnancy BMI. The risk of obesity associated with cesarean delivery has tended to be lower in epidemiological studies that have adjusted for maternal prepregnancy BMI than in studies without this adjustment.^21,38^ Given that prepregnancy BMI is associated with mode of delivery^39^ and overweight or obesity among children^38,40^ and the lack of an association between delivery mode and adiposity in this study and other studies adjusting for maternal BMI,^19,28,29,30^ future epidemiological studies of mode of delivery and body composition and adiposity among offspring should consider this important maternal confounder.^21^

### Strengths and Limitations

This study has strengths. Body composition was measured directly using DXA, and measures were obtained by highly trained staff using standardized protocols. Compared with computed tomography, DXA has been shown to be more reproducible and less costly and to have a lower radiation burden to measure VAT, SAAT and TAAT; thus, it is a more favorable method in studies of children.^41^ In addition, the inclusion of a number of detailed covariates in this study, including maternal prepregnancy BMI, may have reduced the possibility of residual confounding in the associations. Additional strengths include the prospective study design and the use of sophisticated statistical methods, such as stabilized inverse probability weights, to account for selection bias owing to loss to follow-up (even though similar rates of cesarean delivery and maternal-child characteristics were observed between included and excluded participants).

This study also has limitations. Because participating women were enrolled from private obstetric practices, they may have had higher levels of education and income than women in the general population, possibly limiting generalizability. Also, use of self-reported maternal prepregnancy weight and height to calculate maternal BMI is prone to measurement error. However, a previous study^42^ reported a correlation (*r*, 0.99) between self-reported and clinical prepregnancy weight in a subset of 343 Project Viva participants. Although the sample size for the main analysis (cesarean vs vaginal delivery) allowed for sufficient statistical power, the sample size was limited for data analyses in which cesarean deliveries were further classified according to indication and timing in relation to onset of labor.

## Conclusions

In this cohort study, adolescents born by cesarean delivery had significantly higher measures of lean mass, fat mass, and central adiposity compared with those born by vaginal delivery, but associations did not remain after adjustment for the mothers’ self-reported prepregnancy BMI. These findings suggest that the association between birth by cesarean delivery and adolescent adiposity may partly be explained by maternal self-reported prepregnancy BMI. In addition, the associations between cesarean delivery and measures of lean and fat mass in offspring differed when classifying cesarean deliveries by their timing in relation to labor. Future studies evaluating the association between birth by cesarean delivery and childhood adiposity may benefit from using direct measures of body composition rather than anthropometric measures as primary study outcomes, obtaining detailed information on the circumstances of cesarean delivery, and incorporating relevant prepregnancy information including maternal prepregnancy BMI.

## Notes

Supplement. **eTable.** Characteristics of mother-child pairs included vs excluded from the current analysis in Project Viva
