# Supplementary material for: Association of Mode of Obstetric Delivery With Child and Adolescent Body Composition
Source: JAMA Netw Open. 2021 Oct 8;4(10):e2125161. doi: 10.1001/jamanetworkopen.2021.25161 (PMC8501392; doi:10.1001/jamanetworkopen.2021.25161)
Supplement: Supplement. — eTable. Characteristics of mother-child pairs included vs excluded from the current analysis in Project Viva [file jamanetwopen-e2125161-s001.pdf]

Supplementary Online Content

Mínguez-Alarcón L, Rifas-Shiman SL, Sordillo JE, et al. Association of mode of obstetric delivery with child and adolescent body composition. *JAMA Netw Open*. 2021;4(10):e2125161. doi:10.1001/jamanetworkopen.2021.25161

eTable. Characteristics of mother-child pairs included vs excluded from the current analysis in Project Viva

This supplementary material has been provided by the authors to give readers additional information about their work.

© 2021 Mínguez-Alarcón L et al. *JAMA Network Open*.

eTable 1. Characteristics of mother-child pairs included vs excluded from the current analysis in Project Viva

	**Total Viva Cohort**	**Included**	**Excluded**
	N=2128	n=975	n=1153
**Mother**			
Mode of delivery, %			
Vaginal	1600 (76)	763 (78)	837 (75)
Cesarean	498 (24)	212 (22)	286 (25)
Age, years	31.8 (5.2)	32.0 (5.5)	31.7 (5.0)
Pre-pregnancy BMI, kg/m^2	24.9 (5.5)	25.0 (5.4)	24.8 (5.7)
Total GWG, kg	15.5 (5.7)	15.4 (5.5)	15.6 (5.9)
Race/ethnicity, %			
Black	348 (17)	175 (18)	173 (15)
Hispanic	154 (7)	65 (7)	89 (8)
White	1399 (66)	631 (65)	768 (68)
Other	203 (10)	98 (10)	105 (9)
College graduate, %			
No	744 (35)	326 (34)	418 (37)
Yes	1360 (65)	643 (66)	717 (63)
Pregnancy smoking status, %			
Never	1443 (68)	694 (71)	749 (66)
Former	398 (19)	180 (19)	218 (19)
Current	266 (13)	97 (10)	169 (15)
Father's BMI, kg/m^2	26.4 (4.1)	26.5 (4.0)	26.4 (4.2)
Child sex			
Male	1096 (52)	484 (50)	612 (53)
Female	1032 (48)	491 (50)	541 (47)
Birthweight for GA z-score	0.17 (0.97)	0.19 (0.98)	0.16 (0.96)

We presented data as mean (SD) unless stated otherwise.

© 2021 Mínguez-Alarcón L et al. *JAMA Network Open*.
